# Effect of hierarchically aligned fibrin hydrogel in regeneration of spinal cord injury demonstrated by tractography: A pilot study

**DOI:** 10.1038/srep40017

**Published:** 2017-01-09

**Authors:** Zhenxia Zhang, Shenglian Yao, Sheng Xie, Xiumei Wang, Feiyan Chang, Jie Luo, Jingming Wang, Jun Fu

**Affiliations:** 1 Department of Radiology, Peking University China-Japan Friendship School of Clinical Medicine, BeiJing, 100029, China; 2 School of Materials Science and Engineering, Tsinghua University, BeiJing, 100084, China; 3 Department of Radiology, China-Japan Friendship Hospital, BeiJing, 100029, China; 4 Department of Pathology, China-Japan Friendship Hospital, BeiJing, 100029, China; 5 Department of orthopedics, PLA General Hospital, BeiJing, 100853, China

## Abstract

Some studies have reported that scaffold or cell-based transplantation may improve functional recovery following SCI, but no imaging information regarding regeneration has been provided to date. This study used tractography to show the regenerating process induced by a new biomaterial-aligned fibrin hydrogel (AFG). A total of eight canines subjected to SCI procedures were assigned to the control or the AFG group. AFG was implanted into the SCI lesion immediately after injury in 5 canines. A follow-up was performed at 12 weeks to evaluate the therapeutic effect including the hindlimb functional recovery, anisotropy and continuity of fibers on tractography. Using tractography, we found new fibers running across the SCI in three canines of the AFG group. Further histological examination confirmed limited glial scarring and regenerated nerve fibers in the lesions. Moreover, Repeated Measures Analysis revealed a significantly different change in fractional anisotropy (FA) between the two groups during the follow-up interval. An increase in FA during the post injury time interval was detected in the AFG group, indicating a beneficial effect of AFG in the rehabilitation of injured axons. Using tractography, AFG was suggested to be helpful in the restoration of fibers in SCI lesions, thus leading to promoted functional recovery.

The incidence of spinal cord injuries (SCI) is increasing all over the world, with thousands of new SCI cases in the world annually^1^. SCI is often devastating and irreversible, resulting in chronic neuropathic pain, partial or complete paralysis. The major pathological findings in spinal cord injury include the interruption of ascending and descending axonal pathways, loss of neurons and astrocytic proliferation, inflammation, and demyelination. Deficits in neurologic function below the level of SCI are thought to be mostly due to the loss of white matter in and around the injury site^2^.

There are very few treatments available to improve the outcomes of spinal cord injuries. Promoting axonal regeneration is considered a potential repair strategy because it may lead to the recovery of axonal circuits involved in motor and/or sensory function. The central nervous system (CNS) neurons are intrinsically capable of regenerating damaged axons to a certain degree, but their attempts after SCI are hindered by structural and chemical obstructions in the damaged nervous tissue, such as derangements in ionic homeostasis, accumulation of neurotransmitters, free-radical production, astroglial scar launch, immune cell invasion and the release of cytokines^3^. The application of synthetic and natural biomaterials to modify the growth-inhibitory terrain in the injured spinal cord is potentially helpful in eliciting axonal regeneration and fostering functional restoration. Austin *et al*. have shown that a hydrogel of hyaluronan and methyl cellulose (HAMC) is capable of modulating inflammation, axonal preservation and scarring events in a rodent model, leading to improved functional recovery following severe SCI^4^. Another recent study has shown that implants of linear-ordered collagen scaffold in combination with collagen-binding, domain-brain-derived neurotrophic factor strikingly improved the locomotion and functional sensory recovery on completely transected SCI models, rendering some canines capable of standing unassisted and transiently moving. Their histological analysis showed that administration of biomaterial implants reduced lesion volume, decreased collagen deposits, promoted axon regeneration and improved myelination^5^. However, no imaging details documenting the process of remodeling and reconstruction of the spinal cord were provided in their study.

Diffusion tensor imaging (DTI) is an advanced technique of DWI that offers the possibility to track and graphically depict axonal fiber bundles by tractography^6^,^7^,^8^,^9^,^10^. DTI with subsequent fiber tracking is capable of visualizing the white matter, and this technical advancement enables the reconstruction of white matter tracts in 3D view, not only of the brain but also of the spinal cord. DTI tractography can be used as a qualitative indicator of SCI to track the damaged nerve fibers visually and clearly observe the axonal bundles lesion^9^,^11^,^12^,^13^. A previous study has shown that DTI tractography demonstrated the disruption of rubrospinal tract axons while indicating which axon tracts were preserved. The authors thought that DTI may be able to both delineate the location and number of surviving axons following spinal cord injury^10^. Brian *et al*. have established a traumatic spinal cord injury rat model and their results of locomotor analyses and histopathologic evaluations revealed positive correlations between DTI imaging (FA values), locomotor activity (BBB score), and spared tissue measurements^12^.

In the present work, we established a spinal cord hemisection injury canine model and transplanted into lateral hemisection SCI with hierarchically aligned fibrin hydrogels (AFG), which has great promise not only in construction of 3D aligned microtissue *in vitro*, but also in promotion of neural regeneration in the central nervous system *in vivo*^14^. We combined DTI with subsequent fiber tracking, histopathologic evaluations and locomotor analyses to evaluate the changes of the SCI within twelve postoperative weeks. Our hypothesis is that the AFG may exert great effects on the recovery of spinal cord white matter injury and that DTI can provide information statistically and visually to assess the integrity of spinal cord fibers, which would be in line with histopathologic results.

## Materials and Methods

### Preparation of fibrin hydrogels

**Figure 1:**
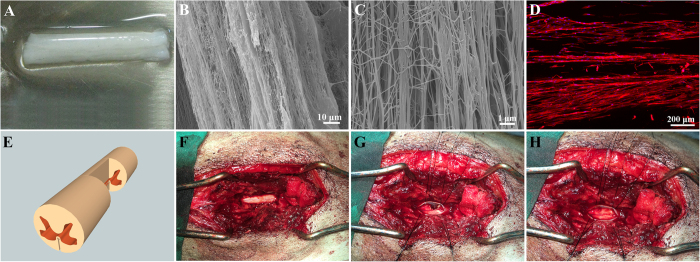
Aligned Fibrin hydrogel (AFG) scaffold and canine hemisected spinal cord injury model. Macro-photo (**A**) and micrograph (**B**,**C**) taken by scanning electron microscope show network architecture of AFG, the nanofiber. Longitudinal section of AFG scaffold demonstrating the aligned nanoscale fibrous structure (**B**,**C**). hMSCs cultured on the AFG scaffold for 3 days (red was f-actin and blue was nucleus) (**D**). Schematic diagram of the hemisection model (**E**). AFG scaffold implantation procedure included exposing of the spinal cord (**F**), hemisection (**G**) and filling of the AFG scaffold into the defect (**H**).

Fibrinogen, thrombin, and polyethylene glycol (PEO, average MW ca. 4000 kDa) were purchased from Sigma Aldrich. Hierarchical AFG was prepared using a modified electrospinning method reported in a previous study. In brief, 10 mg/mL fibrinogen solution in saline with 0.5% PEO was electrospun and collected using a liquid bath with 50 mM of CaCl_2_ and 5–10 units/mL of thrombin. The structure of the material is presented in Fig. 1.

### Animals and modeling procedure

All animal protocols were approved by the recommendations of the Animal Ethics Committee of Peking University (Grant No. LA201515) (Beijing, China). The study was carried out in strict accordance with the approved guidelines. A total of eight healthy 2–3-year-old male mongrel canines, weighing an average of 15 kg, were enrolled in the present study. All the canines were neurologically normal and clinically determined to be in good health. Three canines were allocated into the control group and 5 were assigned to the AFG group. Anesthesia was performed using the pentobarbital sodium intravenous injection, 30 mg/kg. The hair on the back was shaved and the skin was sterilized using povidone iodine. Dorsal laminectomy was performed at L2–L3 level using bone rongeurs and microscissors under sterile conditions. The dura was opened with a surgical blade to expose about 1.5 cm of the spinal cord. A 5 mm segment of the lateral lumbar spinal cord was removed by hemisection. A piece of AFG was implanted into the lesion cavity immediately after injury in each canine of the AFG group, while the lesions in the control group were filled with saline. The process of the procedure is presented in Fig. 1. Finally, a collagen membrane was placed over the exposed spinal cord covering the edges of the dura to prevent peridural adhesion and scar formation. After surgery, all canines were immediately administered an intravenous infusion of saline solution and injected with penicillin twice daily for 3 days to prevent infection. The canines were raised as a closed herd and kept under a strict quarantine protocol. Principles of laboratory animal care were followed and every effort was made to minimize animal suffering.

### MRI procedure

The baseline MR examinations were performed on canines before surgery. Follow-up MR examinations were conducted in all canines at the following time points: 1 week, 3 weeks, 6 weeks, 9 weeks and 12 weeks postoperatively. All MRI data were acquired using a 3.0 T whole-body MRI scanner (Ingenia, Philips Medical Systems, Best, The Netherlands) with 15-channel SENSE (sensitivity encoding)-spine-coil. First, coronal, sagittal and axial T2-weighted images were obtained with turbo spin-echo sequences. Axial plane parameters were as follows: TR/TE 3571/120 ms, FOV 122 mm (AP) × 81 mm (FH) × 160 (RL) mm; matrix 200 × 152; slice thickness 3.0 mm with a 0.4 mm inter-slice gap; 24 slices; and the number of signal average equaled 3. Sagittal and coronal plane parameters were as follows: TR/TE 2500/113 ms; FOV 180 mm (AP) × 360 mm (FH) × 270 mm (RL); matrix 300 × 564; slice thickness 3 mm with a 1.0 mm inter-slice gap; 7 slices; and the number of signal average equaled 4. Then DTI was acquired with a single-shot echo planar imaging sequence with two b values (b = 0 and 500 s/mm^2^). Diffusion-sensitizing gradients were applied along 15 noncollinear directions. Forty contiguous axial slices were acquired with 3 mm thickness and no gap. The acquisition parameters were as follows: TR/TE 4781/64 ms; FOV 240 mm × 240 mm; matrix 108 × 66 (M × P) with a reconstruction matrix of 224 × 128; flip angle 90°; voxel size 1.5 mm × 1.5 mm × 3.0 mm; the number of signal average equaled 6. Saturation bands were set on the canine’s chest and abdomen to reduce movement artifacts. The prescription of the scanning center and scope was maintained to be consistent across all the scans for each canine.

### DTI data analysis

**Figure 2:**
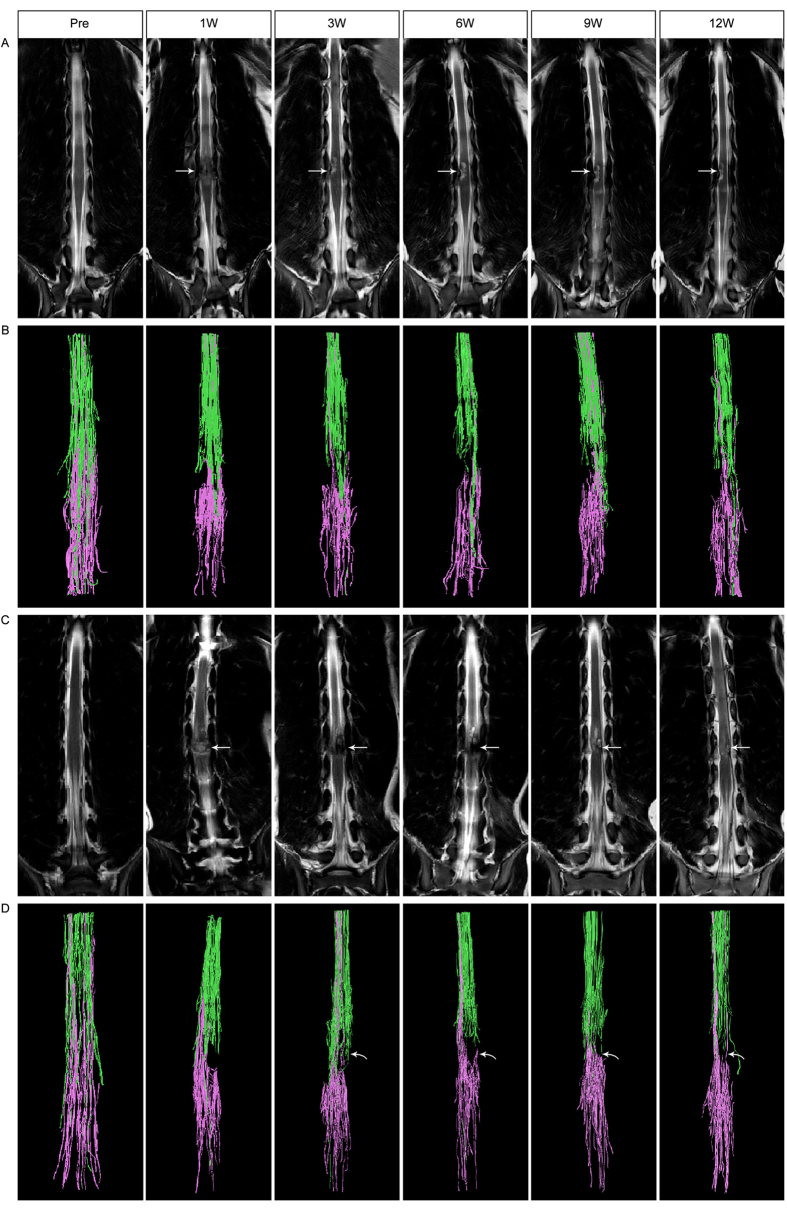
Time-dependent changes of spinal cord on T2 weighted and tractography images. Extensive necrosis and cystic formation in the spinal cord are demonstrated on coronal T2 weighted images in a control dog (Row **A**), whereas the lesion secondary to SCI is reduced in size due to the implantation of AFG in Canine 2 of AFG group (arrow, Row **C**). Fiber tractography images of the spinal cord before and after SCI for the control dog (Row **B**) and Canine 2 (AFG group, Row **D**) depict the regeneration of fibers post injury in Canine 2 (curved arrows) and discontinuity of fibers in the control dog. Green stands for fibers traced from the ROI rostral to the epicenter, where purple corresponds to fibers traced from the ROI caudal to the epicenter.

The DTI data were transferred to an Extended MR workspace (Version 2.6.3.5 HF 3 2013, Philips Medical Systems) for postprocessing. T2W images were fused with DTI images to identify the spinal cord in axial and craniocaudal directions. First, two “seed” ROIs were placed 5 mm rostral and 20 mm rostral to the lesion epicenter, respectively. With the threshold FA value set to 0.3, the fibers were traced downwards (shown in green) using the Deterministic tractography algorithm^15^, and the FA values of these traced antegrade fibers were obtained for statistical analysis. The FA values at the injury epicenter were too low to permit tracking through or around them; however, the images suggested fibers passing through the preserved tissue surrounding the syrinx, although they could not be followed into the continuing cord segment. To overcome this limitation, an additional seed point was set caudal to the SCI site and the fibers were traced upwards (shown in purple). Thus the whole outline of fibers in the spinal cord was depicted, with fibers appearing to meet within the tissue surrounding the syrinx, suggesting continuity (Fig. 2).

### Functional recovery assessment

An independent researcher video-recorded the hindlimb movements of all the injured canines before surgery and at week 1, 3, 6, 9, 12 after surgery. Functional recovery was evaluated according to the BBB scoring system^16^, by two other independent researchers. During the assessment, the canines moved freely in an open field and were rated on the basis of their ability for spontaneous or voluntary hindlimb motion.

### Histological examination

Twelve weeks after surgery, the segments of SCI were retrieved and fixed in 4% formaldehyde for 48 h, embedded in paraffin, and cut into 5-um thick sections (Leica Microsystems). The analysis of injured spinal cord was performed on longitudinal sections. Continuous tissue sections were stained with hematoxylin and eosin (H&E) for general observation of cellular and extracellular matrix features. Masson’s trichrome staining (MTS) was used to identify the presence of myelin sheath (neurogenic tissue) within the defect. Masson’s trichrome is not only able to show myelin in the nervous tissue, but also depict collagenous tissue within the defect. All histological pictures were taken under Leica SCN400 Slide Scanner (Leica Microsystems, Germany). In addition, immunofluorescence staining for Neurofilament-160 (NF-160) and Growth-associated protein-43 (GAP-43) were carried out to show the axons and their regeneration. The immunostained cells were visualized under fluorescence microscope (Zeiss LSM 780, Germany) with a color digital camera.

### Statistical analysis

Repeated Measures Analysis were performed to examine the changes in FA and BBB scores during the post injury time interval and the differences in these parameters between groups, and repeatability of FA measurement was determined by calculating the intraclass correlation coefficient (ICC). All statistical analyses were carried out using the SPSS 16.0 software (SPSS Inc., Chicago, Illinois, USA).

## Results

### Conventional T2 weighted imaging

One week after the SCI, the injured spinal cord showed high signal intensity on T2 weighted MRI, with prominent edema and mild to moderate hemorrhage. The borders of the injury became distinctive at 6 weeks postoperatively, indicating the presence of necrosis in the lesions. At 12 weeks post injury, the final extent of injury varied among the canines, from 6–14 mm in the controls to 5–12 mm in the AFG group. Cystic formation was detected in all canines at the end of follow-up (see Fig. 2).

### Quantification of fibers in canine

Tractography depicted intact neural fibers in the spinal cord of the canines before the injury. At 1–3 weeks after SCI, the nerve fiber bundles in all injured sites were completely disrupted, showing a defect between the fibers traced antegrade and retrograde, while those on the contralateral side continued caudally (Fig. 2B,D). At 6 weeks and 12 weeks postoperatively, there were a few fibers running across the SCI side in canine 2 (Fig. 2D), 4 and canine 8 of the AFG group, though the fiber bundles were slim and deformed to some extent. Although no newborn fibers were observed in canine 5 and canine 7, the elongation of traced bundles was discovered on the hemisection side. In contrast, progressive deterioration of lesions occurred in the control group, with a greater degree of disruption of the fibers at the chronic stage (Fig. 2B).

Measurements of FA were repeatable in our study, with ICC of 0.901. Sphericity assumption has been confirmed by the Mauchley’s Test (*P* = 0.287). The Repeated Measures Analysis revealed a significant effect of time (*P* < 0.001), which indicated significant FA changes of the injuried spinal cord during the post injury time interval. In addition, “time × group” interaction on FA was detected (Sphericity Assumed, *P* < 0.001), suggesting different temporal changes in FA between the groups. Mann-Whitney Test detected a significant difference in FA between them at 9 weeks (*P* = 0.025) and 12 weeks (*P* = 0.024) post injury, respectively. From the graph, it was evident that the average FA values of traced antegrade fibers increased consistently after 3 weeks post injury in the AFG group, while such a trend was absent in the control group. Regarding the BBB scores, only the time effect was found, while no group effect or interaction was statistically significant.

### Functional recovery in canine SCI

**Figure 3:**
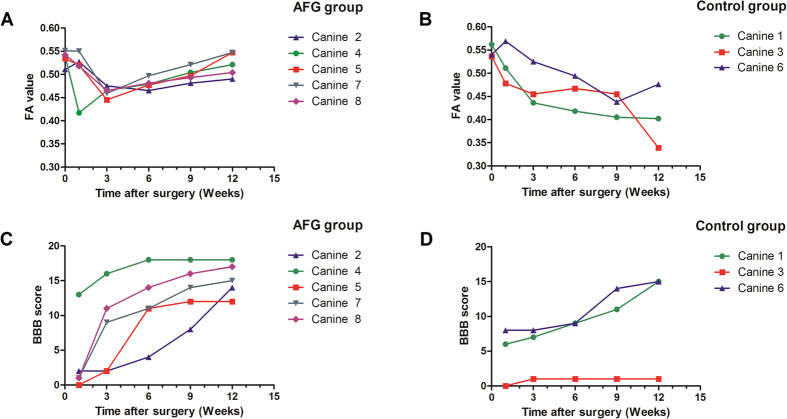
FA values and BBB scores of the AFG group (**A,C**) and control group (**B,D**) were plotted at different time points.

**Figure 4:**
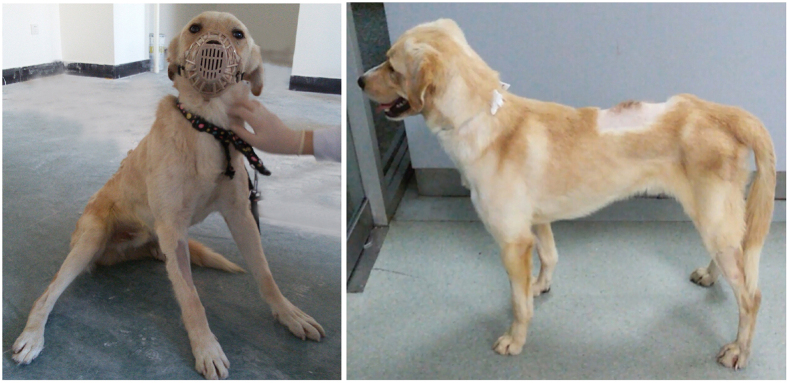
Photos of Canine 2 in AFG group. She suffered from paralysis in her left hind limbs at week 1 post injury (left), but with the treatment of AFG, she gained behavioral function recovery at week 12 post injury (right).

The BBB scores of the two groups recorded throughout the experiment are shown in Fig. 3. Immediately after surgery, all canines showed paralysis in all ipsilateral limbs with a BBB score of 0–13. In the control group, injured canines had very limited self-healing and motor function. In contrast, the canines with AFG biomaterial treatment have shown persistent locomotion recovery in the 12-week observation period and achieved a stable BBB score of approximately 12–18 at week 12, which corresponded to weight bearing, and forelimb- hindlimb coordination (Fig. 3). These results have been recorded in supplementary videos. Figure 4 shows a canine with AFG biomaterial treatment achieved persistent weight bearing with the hindlimb ipsilateral to SCI at week 12 post injury.

### Histological changes after spinal cord injury

**Figure 5:**
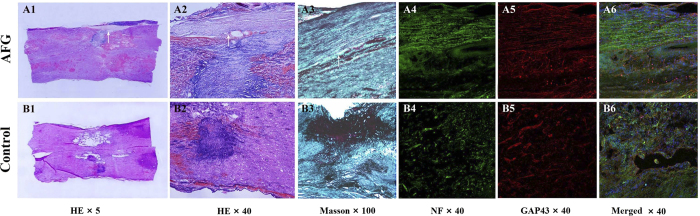
Histological photographs of spinal cord lesions in Canine 2 (upper row) and a control dog (lower row). HE staining coronal sections show general structural changes of the spinal cord tissue after SCI. Extensive necrosis and prominent cavitation boundary to the scar predominates at the epicenter in the control dog (**B1,B2**), while they are much less appreciable in Canine 2 of the AFG group (**A1,A2**). Regenerated, aligned fibers are noted adjacent to the scar (arrow) in Canine 2. Masson staining also displays the regular arrangements of myelinated nerve fibers (**A3**), in contrast to the heavy collagen deposits by the green staining in the Control (**B3**). Immunofluorescence for NF-160 (green color) and GAP-43 (red color) shows positive expression of neurofilament-160 on the axons (**A4**) and growth-associated protein on the axons (**A5**) in canine 2, demonstrating the regeneration of the fibers. Scattered and slight expression of NF-160 is seen in the section of the control dog (**B4**), but no presence of positive expression of GAP-43 is found (**B5**). The merged images are presented as **A6** and **B6**.

Both H&E and Masson staining of the samples showed prominent damage in the spinal cord at the lesion epicenter. We observed large volumes of scars and disordered structures in the control group, and there were degenerative neurons in the spinal cord, as well as nerve fiber tumefaction, numerous vacuoles, myelin sheath lamellar separation, degeneration, and proliferation of glial cells in the control group. Upon magnification of the injury epicenter, severe fibroblast-like cell proliferation was detected by Masson staining in the control group as a result of chronic inflammation. In contrast, less severe pathological changes in the parenchyma were detected at the epicenter in the AFG group, and a great reduction of fibrosis and degradation was observed compared with the controls. The growth of regenerative fascicular nerve fibers was observed in the AFG group (Fig. 5A1). In canines 2, 4 and 8 of the AFG group, regenerated axons were noted adjacent to the scar tissue, which corresponded to the newborn traced fibers on tractography. It is of note that the immunofluorecence staining showed regenerative fibers adjacent to the scar (Fig. 5A5). In canine 5 and canine 7, mild demyelination of fibers was observed at the boundary of the epicenter, close to the scar.

## Discussion

As confirmed by histological observation, the effects of therapy with AFG implantation into the spinal cord were demonstrated in the canine SCI models using tractography. The physical blend of AFG reduced the extent of scarring and inflammation in the SCI lesion. Moreover, this biomaterial acted as a guide in the regeneration of new fibers. Regeneration of nerve fibers was detected on histological sections, which was in agreement with the reconstruction of fibers in the hemisection side on tractography. Long-term follow-up revealed encouraging improvement of hindlimb locomotion in the AFG group.

AFG, prepared by a modified electrospinning technique with a concurrent molecular self-assembly process, is a type of cell- and regeneration-activating biomaterial that creates an artificial micro-environment suitable for axonal regeneration. The nature of the self-assembly process would allow formation of cellular wires *in situ* that have any length for use in biological applications. Because of its biomimetic hierarchical structures, this novel biomaterial has a good biocompatibility and bioactivity. It has been reported that 3D aligned nanofibers designed specifically to mimic the native architecture of nerve were helpful to support neural cell growth and function^17^,^18^,^19^. Scaffolds encapsulating neural progenitor cells were formed *in situ* within the spinal cord and resulted in the growth of oriented processes *in vivo*^17^. Schwann cells were demonstrated to adhere to and proliferate in aligned nanofibers with great efficacy, and histological examinations also demonstrated increased axonal and schwann cell regeneration within the reconstructed nerve gap in animals^18^. Similarly, the excellent biocompatibility and cellular active sites of AFG offer the potential to bridge the cavities and provide a favorable environment during the process of axonal regeneration. Our results indicate that the implantation of AFG reduced the extent of injury and enhanced functional recovery in SCI canines. Masson staining indicated that AFG reduced fibrous scarring, which might have been able to promote endogenous regeneration due to decreased chondroitin sulfate proteoglycans expression^4^. The hindlimb of the SCI side of canine 2 and 8 finally achieved consistent weight-bearing at week 12 post injury. This was an amazing finding since the canine had been paralyzed in the 3 weeks post injury. A deformed newborn fiber was discovered on tractography and upon histological section, which was considered to be a part of the descending motor pathway and critical to functional recovery. Immunofluorescence staining further confirmed regeneration of axons. Canines have a corticospinal tract in the lateral column of the spinal cord, suggesting that the physiologic basis underlying locomotor recovery in canine species is closer to humans than rodents^20^. The ability to preserve weight-bearing has significant clinical implications for patients with SCI through reduction of associated comorbidities, such as pulmonary infections and skin breakdown.

DTI tractography can be used as a qualitative indicator of SCI to track the damaged nerve fibers visually and observe the lesion of axonal bundles clearly^9^,^11^,^12^,^21^,^22^,^23^. Recent studies indicate that quantitative DTI parameters are sensitive and specific biomarkers of spinal cord white matter integrity^21^,^22^,^23^. Our data revealed a disruption of fibers on the hemisection side after the SCI, as well as regeneration of tract fibers at the chronic stage in canine 2, 4 and 8 of the AFG group. The findings on tractography were in accordance with the motor performance and histological findings in our study. Previous studies have reported that injured spinal cords show a decrease in anisotropy, resulting from a disruption of longitudinally aligned axons and also exhibited a decrease in FA^23^,^24^,^25^. In this study, we focused on tractography to visualize the configuration of traced fibers. Meanwhile, the FA values of the traced antegrade fibers were extracted to indicate the integrity of the epicenter white matter. Residual fibers had lower anisotropy than normal fibers for a number of reasons including axonal degeneration, demyelination, disintegration along the longitudinal axis, and widespread deterioration of the microenvironment^11^,^26^. We examined the fibers rostral to the epicenter, since the relative health of preserved white matter fibers after SCI may be predictive of potential recovery. Studies examining the mechanism and therapeutic interventions, which aimed at attenuating axonal growth inhibition after experimental SCI, indicated the importance of perilesional axon health via preservation and growth of remaining fibers past the lesion sites^27^. On the other hand, it was difficult to examine the FA and ADC of the injured sites because of poor reproducibility^24^. Good reproducibility and reliable data were obtained using tractography in our study. Consistent with previous studies, the regeneration and reorganization of fibers resulted in an increase of FA in our AFG group. The FA values at week 1 post injury were abnormally high in some canines, which was inconsistent with other data showing the lowest FA values in the postoperative acute phase^23^,^24^,^25^,^28^,^29^,^30^. This phenomenon has been reported before, but was not clearly explained^6^,^31^. There are possible reasons regarding the presence of high FA in the acute phase. If the extracellular water diffusivity is more restricted on the axial plane, while the preserved axons maintain relatively normal diffusivity on the longitudinal direction, it will result in an increase in FA. More than 3 weeks post injury, the quantitative measures suggested a gradual improvement, which indicated that AFG had a beneficial effect in the reconstruction of perilesional white matter integrity. However, it should be noted that tractography methods can produce false positive and false negative results. The measured diffusion effects are averaged over a voxel, complicating the biophysical interpretation of the diffusion tensor^32^,^33^. Fortunately, the fiber tracts in the spinal cord are longitudinal, which avoid the tractography error resulting from the crossing fibers in a single voxel. There are also some argues regarding the meanings of FA changes. For example, in scientific studies FA is often interpreted as “white matter integrity,” however many factors (e.g. cell death, change in myelination, increase in extracellular or intracellular water, etc.) may cause changes in FA. In our study, the longitudinal increases of FA can be attributed to the regenerative effect, as well as reorganization. However, there is still difficulty in interpretation of DTI due to the fact that the scale at which diffusion is measured with DTI is very different from the size scale of individual axons.

Unexpectedly, canine 5 and 7 in the AFG group failed to show any regenerated fiber on tractography at the end of the follow-up. However, formation of a new fiber was observed on tractography at week 6, which disappeared in subsequent examinations. Notably, the newborn fibers running across the injured sites appeared at week 6 post injury in canine 2 (Fig. 2D), but showed disruption or slim at week 9 post injury as well. This phenomenon may be attributed to secondary injury in SCI. It was at week 6–9 post injury that we noticed distinct cystic formation in the SCI lesions. The process of progressive central cavitation adds to the complexity of regenerative failure, leading to scar-encapsulated cavity many times the size of the initial lesion. The physical process of cavitation leads to astrocyte abandonment of neuronal processes, neurite stretching, and secondary injury. Although AFG acted as the architecture skeleton in the initial stage, it self-dissolved and lost its supportive effect 2–3 weeks after implantation. Therefore, severe secondary injury may interrupt the regeneration process and counteract therapeutic interventions.

Whether or not the FA showed an appreciable increase after the SCI, the overall hindlimb function underwent recovery in all canines, with more prominent recovery in the AFG group. Studies of SCI in animal models offer interesting perspectives on this very general neurobiological problem of functional recovery after CNS lesion, especially in the context of locomotion. The pioneering work of Grillner firmly established the concept of central generator pattern, showing that it was innate^34^. Adult chronic spinal cats could regain spontaneous hindlimb locomotion on a treadmill after weeks of locomotor training^35^,^36^ and that this recovery could be accelerated by intensive training with daily injections of clonidine^37^. It is observed that large lesions of descending pathways do not prevent functional recovery of voluntary quadrupedal locomotion even if some deficits persist over time. A previous study deduced that after partial spinal injuries, the expression of the hindlimb locomotor pattern could primarily be attributable to the intrinsic reorganization and re-expression of the spinal locomotor CGP below the lesion^38^. A major functional contribution from the spinal cord itself may operate even in the presence of only limited descending inputs. This further highlights the importance of fostering such spinal cord potential of neuroplasticity in rehabilitation strategies in humans with SCI. In our study, the control dogs got functional recovery to some extent as well, which could be attributed to the function of central generator pattern.

There were several limitations in this study. First, this study did not use a model of contusive SCI, which is similar to human traumatic SCI; instead, we used a simpler hemisection model to implant the biomaterial. It is difficult to maintain the consistency in severity of SCI across all canines. In addition to surgical manipulation, various elements including mechanical disruption, extracellular edema, hemorrhage, loss of spatial organization, liquefaction or cystic degeneration led to a varying severity of SCI in these canines. Besides, arachnoid inflammation and scarring related to subdural surgical procedures was present, which may hamper the improvement of functional recovery. Secondly, the sample in our study is limited, and is not sufficient for reliable statistical analysis. More data are needed to further confirm our observation. Thirdly, although the preliminary results have shown the therapeutic effect of AFG in the SCI, the regeneration process was somehow locked by secondary injury, which was not fully examined in this study. We need to design a more precise experiment to study the interaction of the biomaterial and the secondary injury.

In summary, by using consecutive tractography and histological examination, we demonstrated the regeneration of fibers in SCI lesions on canine hemisection SCI models, which might be attributed to the therapeutic effect of AFG. The present study shows AFG may be effective in spinal cord rehabilitation after injury and deserves further investigation.

## Additional Information

**How to cite this article**: Zhang, Z. *et al*. Effect of hierarchically aligned fibrin hydrogel in regeneration of spinal cord injury demonstrated by tractography: A pilot study. *Sci. Rep.* **7**, 40017; doi: 10.1038/srep40017 (2017).

**Publisher's note:** Springer Nature remains neutral with regard to jurisdictional claims in published maps and institutional affiliations.

## Supplementary Material

Supplementary Information

Supplementary Video 1

Supplementary Video 2

Supplementary Video 3

Supplementary Video 4
