# Supplementary material for: Effect of hierarchically aligned fibrin hydrogel in regeneration of spinal cord injury demonstrated by tractography: A pilot study
Source: Sci Rep. 2017 Jan 9;7:40017. doi: 10.1038/srep40017 (PMC5220328; doi:10.1038/srep40017)
Supplement: Supplementary Information [file srep40017-s1.doc]

**Effect of hierarchically aligned fibrin hydrogel in regeneration of spinal cord injury demonstrated by tractography: A pilot study**

**Zhenxia Zhang^1^, Shenglian Yao^2^, Sheng Xie^3*^, Xiumei Wang^2*^, Feiyan Chang^3^, Jie Luo^4^ , Jingming Wang^5^, Jun Fu^5^**

**video legend**

The videos of canine 2 in AFG group at week 3 (video 1) and week 12 (video 2). Video 1 shows slight movement of the limb ipsilateral to spinal cord injury at week 3. Video 2 shows consistent weight-supported plantar steps and frequent forelimb-hindlimb movement coordination.

The videos of canine 3 in control group at week 3 (video 3) and week 12 (video 4). Video 3 and Video 4 show slight movement of hip joint at week 3 and week12.
